# Mast Cells in Early Rheumatoid Arthritis

**DOI:** 10.3390/ijms20082040

**Published:** 2019-04-25

**Authors:** Felice Rivellese, Francesca Wanda Rossi, Maria Rosaria Galdiero, Costantino Pitzalis, Amato de Paulis

**Affiliations:** 1 William Harvey Research Institute and Barts and The London School of Medicine and Dentistry, Queen Mary University of London, London EC1M 6BQ, UK; c.pitzalis@qmul.ac.uk; 2 Department of Translational Medical Sciences (DiSMeT) and Center for Basic and Clinical Immunology Research (CISI), University of Naples Federico II, 80131 Naples, Italy; frawrossi@yahoo.it (F.W.R.); mrgaldiero@libero.it (M.R.G.); depaulis@unina.it (A.d.P.)

**Keywords:** mast cells, arthritis, synovial membrane

## Abstract

Rheumatoid arthritis (RA) is a chronic systemic autoimmune disease characterized by inflammation of the synovial membrane, with thickening of the synovial layer, cellular hyperplasia, and infiltration of immune cells. Mast cells (MCs) are cells of the innate immunity present in healthy synovia and part of the cellular hyperplasia characterizing RA synovitis. Although their presence in synovia has been well described, the exact functions and the correlation of MCs with disease development and progression have been debated, particularly because of contradictory data obtained in animal models and from patients with longstanding disease. Here, we present a revision of the literature on MCs in RA, including the most recent observations obtained from patients with early RA, indicating MCs as relevant markers of disease severity in early RA.

## 1. Synovial Inflammation in Rheumatoid Arthritis

Rheumatoid arthritis (RA) is a chronic systemic autoimmune disease primarily affecting the joints [1]. The inflammation of the synovial membrane, i.e., synovitis, is characterized by thickening of the synovial layer, with cellular hyperplasia and infiltration of various type of immune cells, including cells of the innate and adaptive immunity, such as monocytes-macrophages, B and T cells, NK cells and, last but not least, mast cells (MCs) [2]. Many of these immune cells and their mediators, known to be relevant in the pathogenesis of RA [3], have become target of highly specific therapeutic options, directed against mediators (e.g., TNF-alpha, IL-6), immune cells (e.g., CD20 B cells), and, more recently, intracellular signaling pathways (e.g., Jak-Stat pathway inhibitors) [4]. These targeted treatments have revolutionized the approach to RA, leading for the first time in decades to a shift in the paradigm from considering RA as a chronic disease almost inexorably leading to disability to a disease that can be controlled by the induction of remission [5]. Nonetheless, in many patients, the disease progresses because of treatment failure, as up to 40% of patients do not respond to targeted treatment used as first, second, or even third line strategies. To date, despite the progresses in our understanding of the pathogenesis and the clinical features of RA [6], none of the proposed serum biomarkers have proven useful in predicting treatment response, which remains one of the main unmet needs in the field [7]. The disappointment with respect to serum biomarkers includes the well-characterized anti-citrullinated protein antibodies (ACPAs), which have become an invaluable diagnostic tool to identify a subset of patients with worse prognostic features [8] but are of no use in the prediction of treatment response [9].

Therefore, in recent years, there has been a strong interest in the study of the synovial membrane as a tool for patient stratification. The development of minimally invasive techniques, such as US-guided synovial biopsies, has allowed for the analysis of the synovial membrane in patients with early RA and, very importantly, from small joints (e.g., metacarpophalangeal or wrists). This is in clear contrast with previous studies using synovial tissues obtained from joint replacement surgery or by arthroscopy, therefore biased by disease duration and treatment and by the analysis of large joints.

By studying the synovial tissue in large cohort of patients with early untreated RA, a marked heterogeneity in the infiltration of immune cells has been described. More specifically, three distinct histological patterns have been identified: (1) lympho-myeloid dominated by lymphoid lineage infiltration (T cells, B cells, plasma cells) in addition to myeloid cells, (2) diffuse-myeloid with myeloid lineage predominance but poor in B cells/plasma cells, and (3) pauci-immune characterized by scanty immune cells and prevalent stromal cells [2]. Interestingly, the recent analysis of a large cohort (144) of early RA patients naïve to treatment with steroids and immune-suppressors has shown an association between the lympho-myeloid pathotype and disease severity, autoantibody positivity, and, most importantly, radiographic progression [10].

Within the heterogeneous synovial infiltrate of lymphocytes and macrophages, MCs have also emerged as immune cells relevant in the pathogenesis of RA and with potential predictive implications in terms of disease progression and treatment response.

## 2. Emerging Role of Mast Cells in Synovial Inflammation

Tissue MCs and circulating basophils are the only cells that express the high affinity receptor for IgE (FcεRI) and synthesize histamine in humans, although they differ morphologically and immunologically [11,12]. These cells are mostly known as effector cells in allergic reactions in response to antigens and superantigens [13], the mechanisms which account for their functions in allergic diseases [14,15]. Compelling evidence indicates that MCs are involved in several pathophysiological processes, including barrier homeostasis [16], angiogenesis and lymphangiogenesis [17,18], wound healing [19], and tumorigenesis [20,21,22]. MCs have been known to be present in synovia for a long time, but their relevance in the pathogenesis of RA has been somehow neglected, mainly because of contradictory data coming from animal studies and the scarceness of studies in humans [23]. In the early 1990s, our group published the first characterization of synovial human MCs, providing a detailed description of the ultrastructural [24] and functional [25] features of synovial MCs. In parallel, other groups described an increase of MC numbers in the synovia of RA patients, as summarized in Table 1 and described in detail in Section 3.5. However, the association of MCs with clinical features of disease activity has been inconsistently reported, most likely because of the bias coming from the analysis long standing disease and treatment with immune suppressors. Importantly, MCs have been more recently described in other forms of inflammatory arthritis, such as spondyloarthritis [26] and osteoarthritis [27], suggesting that their presence could be a common feature of synovial inflammation, although their exact function as either effector cells or regulators of the immune response in these various settings is yet to be clarified [28]. Several studies aimed at evaluating their role in animal models of arthritis (reviewed in details in [23]), with the most recent publications confirming their relevance in the early phases of the immune response leading to the development of arthritis [29,30]. In parallel, the analysis of MCs in the synovia of a large cohort of patients with early RA unveiled their association with local inflammation, disease severity, and autoantibody positivity, therefore confirming their relevance in RA [31]. 

## 3. Synovial Mast Cells in Early RA

The unprecedented availability of synovial tissue from a large cohort of patients with early RA naïve to treatment with steroids and immunosuppressors (http://www.peac-mrc.mds.qmul.ac.uk/ [10]) has represented an invaluable tool for the assessment of MCs in RA, allowing the analysis of synovial MCs without the bias of disease duration or treatment [31]. In fact, apart from the few studies mentioned above and summarized in Table 1, there has never been a systematic analysis of MCs in the synovia of a large cohort of patients with RA. This study has demonstrated that MC presence in synovia is associated with (i) synovial inflammation, (ii) lympho-myeloid aggregates, (iii) systemic markers of inflammation (ESR, CRP), (iv) autoantibodies such as ACPAs and RF, and (v) disease activity. Importantly, the concept of heterogeneity of synovial inflammation is relatively recent [2]. Therefore the previous studies exploring MCs in RA, although describing variable levels of MC infiltration in synovia, did not consider the possibility that MC infiltration could actually help stratifying patients, which is the most important concept emerging from the recent studies. In the context of this heterogeneous synovial inflammation, the study of synovial MC heterogeneity has also acquired particular relevance.

### 3.1. Synovial MC Heterogeneity

MCs complete their terminal differentiation in peripheral tissues, so their phenotype can be influenced by the local microenvironment, leading to a marked phenotypic and functional heterogeneity, which has been confirmed by recent transcriptomic analyses both in mice [39] and humans [40]. Classically, two types of MCs have been described in humans, based on the differential expression of serine proteases: MC_TC, expressing both tryptase and chymase; MC_T expressing only tryptase. The MC_TC subtype is predominantly found in skin, the gastrointestinal tract, and conjunctiva, whereas the MC_T subtype is the predominant MC type in the lungs, nose, and sinuses [41].

**Figure 1:**
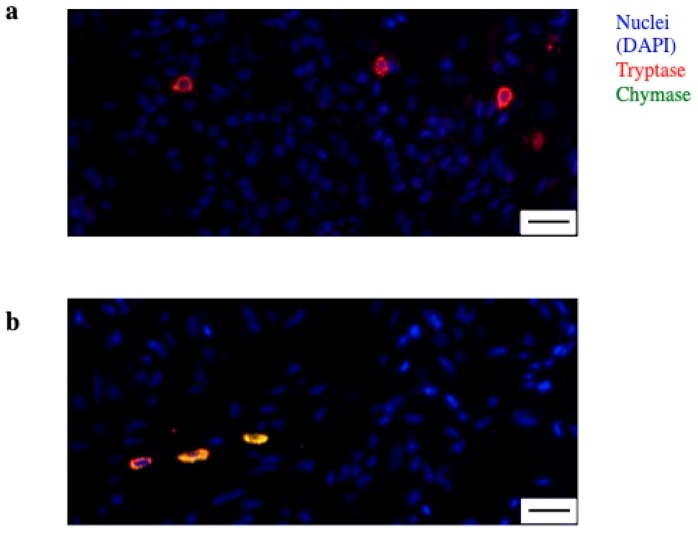
Mast cell (MC) heterogeneity in synovia. (**a**) Immunofluorescence of synovia from an early rheumatoid arthritis (RA) patient showing MC with single positivity for tryptase (red)—MC_T; (**b**) MC double positive for tryptase (red) and chymase (green)—MC_TC. Nuclei in blue (DAPI). Line at 20 µm.

In normal human synovia, over 90% of the MCs are MC_TC [24]. In long-standing RA, an increase of both subtypes has been described, and MC_TC has been shown to be in correlation with disease activity [36]. Another group, however, reported a higher prevalence of MC_T in patients with long-standing RA [35], and in a small cohort of patients with early RA (*n* = 6) a relative increase of the MC_T in correlation with synovial inflammatory score was observed [37]. These data have been expanded by the recent observations coming from a large cohort of patients with early RA, confirming the overall prevalence of MC_TC in RA, with a ratio of 3:1 over MC_T. However, interestingly, MC_T were increased in the synovia of patients with lympho-myeloid infiltrates, in association with disease severity. A summary of these evidences, including details on the heterogeneity of MCs in synovia is offered in Table 1 and an example of MC_T and MC_TC from the synovia of an early RA patient is presented in Figure 1. Overall, although the concept of MC heterogeneity will need to be further explored while keeping into account their plasticity [42], the prevalence of MC_T in association with the disease severity would be in line with the pro-inflammatory role of this subpopulation described in asthma [43].

### 3.2. Synovial Mast Cells as Effector Cells in RA

**Figure 2:**
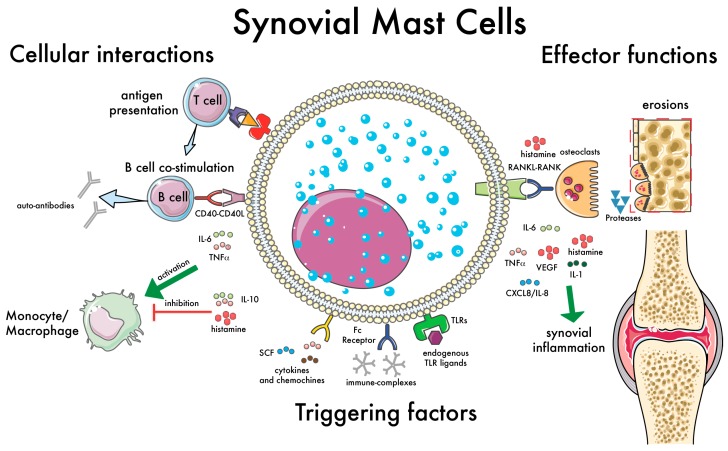
Synovial mast cell (MC): cellular interactions and effector functions. This illustration summarizes the main functions of synovial MCs with relevance in the pathogenesis of RA. Examples of factors capable of triggering MC activation are shown, such as immune complexes [54], cytokines and chemokines [50], and endogenous toll-like receptor (TLR) ligands [48]. The cellular interactions with immune cells are shown on the left: antigen presentation to T cells [55,56]; B cell co-stimulation via CD40–CD40L and soluble mediators (e.g., IL-6), inducing the production of auto-antibodies [31]; modulation of monocyte-macrophages activation [57]. Finally, on the right, the effector functions of MCs are summarized, with an emphasis on the induction of bone erosions, which can be mediated directly by MC-derived proteases [58] or indirectly via the activation of osteoclasts [59,60]. Green arrows indicate the effects of proinflammatory citokines; the “T” bar indicates the effects of antinflammatory citokines.

MCs have the capacity to rapidly perceive immunologic and metabolic insults and initiate different biochemical programs of homeostasis or inflammation. These cells can be activated by a plethora of immunologic and non-immunologic stimuli such as danger associated molecular patterns (DAMPs)-, viral and bacterial proteins [44,45], and endogenous ligands [46]. In the context of RA, specifically, laboratory evidence shows that MCs can produce pro-inflammatory (pro-arthritogenic) molecules [47], while on the other hand many stimuli can activate MCs in vitro, inducting the production of pro-inflammatory mediators. For example, ACPA immune complexes in synergy with toll-like receptor (TLR) ligands have been also shown to induce pro-inflammatory mediators, such as IL-8, from human MCs [48]. Similarly, the activation of synovial MCs has been linked to the production of several pro-inflammatory cytokines, such as TNF-alpha, IL-1beta, and IL-1Ra [49]. Additionally, the synovial fluid possesses MC-chemotactic properties, as it contains stem cell factor (SCF), the main growth and chemotactic factor for MCs [50]. At the same time, the synovial fluid of RA patients also contains several mediators produced by MCs [51]. Finally, MC mediators can contribute to the aberrant survival and activation of human rheumatoid synovial fibroblasts [52]. Importantly, since MCs complete their differentiation in tissues, many of the stimuli known to be present in synovia can contribute to their differentiation. While the exact pathways leading to human MC differentiation from circulating precursors are still debated [53], the two main stimuli needed for human MC differentiation in vitro (SCF and IL-6) are well-known to be present in synovia. Interestingly, higher SCF levels have been described in RA [50], and IL-6 a central cytokine in RA pathogenesis is targeted by biologicaltreatments [3]. The key effector functions of MCs in synovial inflammation are shown in Figure 2. Overall, these data support a pathogenic role as effector cells in RA.

### 3.3. Synovial Mast Cells and the Activation of Lymphocyte

The observation of synovial MCs in association with B and T cell infiltrates in synovia led to the hypothesis that MCs can influence the activation of lymphocytes. Indeed, both mouse and human MCs express MHC class II and can function as antigen presenting cells, inducing antigen-specific T cell activation [55,56,61]. More recently, MCs have also been shown to induce Th17 differentiation via inflammasome-independent IL1β [62]. Overall, the ability of MCs to influence T cell activation is in line with recent data in animal models, showing that in MC-depleted animals the reduced severity of collagen-induced arthritis was also accompanied by a dramatic loss of T cell expansion and reduced T cell cytokine responses [30]. However, while T cells are essential for the break of tolerance and the initiation and perpetuation of the aberrant immune response, B cells also have a well-established role in RA [63,64]. In fact, the presence of B cells in synovia has been linked to local autoantibody production [65,66], osteoclastogenesis/osteoclast activation [67,68], and immune-complex-mediated inflammatory responses [69,70]. Therefore, the possible cross-talk between MCs and B cells is also of relevance in RA. Interestingly, mouse MCs have been described to activate B cells [71,72]. More recently, human MCs were found to induce the activation and differentiation of B cells, including the production of ACPAs [31]. In line with previous data in animal models [71], these effects were found to be dependent on cell–cell contact, with a specific role for CD40L expressed by MCs. We were also able to observe for the first time a direct interaction between human MCs and B cells, as previously shown for mouse MCs and DCs [73] and, very recently, T cells [74]. Overall, these data suggest that MCs, in addition to their established role as effector cells, can influence the adaptive immune response by inducing T and B cell activation, which further confirms their relevance in the induction and progression of autoimmune diseases such as RA. An overview of the key interactions of MCs with immune cells in synovia is presented in Figure 2.

### 3.4. Mast Cells as Immunomodulatory Cells in RA

Convincing evidence, as presented in the previous sections, suggests that MCs can have a prominent role as effector cells in RA and other autoimmune conditions. Nonetheless, MCs are also highly tunable cells, capable of influencing the immune responses towards pro- or anti-inflammatory responses, depending on the type of environment and triggers they are exposed to [75]. For example, in the context of arthritis, when triggered with TLR-ligands and immune complexes, they have been shown to produce pro-inflammatory mediators [48]. However, in response to IL-33 and immune complexes, MCs can also produce immunomodulatory mediators, which in turn dampen the activation of monocytes [57]. These immunomodulatory effects are mediated by preformed mediators, such as histamine, but also newly produced mediators, such as IL-10. Interestingly, histamine is best known as the main pro-inflammatory mediator released by MCs and basophils upon IgE-triggering and is able to mediate the typical symptoms and signs of allergic reactions. However, an increasing amount of evidence suggests that histamine can also induce immunomodulatory and anti-inflammatory effects [76,77]. The potential immunomodulatory functions of MCs in RA are supported by the observation that the serum levels of tryptase and the synovial tryptase mRNA show an inverse correlation with inflammatory markers [78,79]. Overall, there are convincing observations suggesting that MCs can play multifaceted roles in RA. Given the complexity and heterogeneity of RA, however, it is possible to hypothesize that MCs could have different roles in various patients and in the different stages of disease evolution. The only way to confirm this hypothesis is to assess their function systematically, by analyzing their presence in a large cohort of patients at different disease stages and, importantly, in correlation with disease activity. Over the last few years, a number of studies have attempted to tackle the issue of MC presence in the synovia of RA patients, yielding contrasting results that we will review in the next section.

### 3.5. Synovial Mast Cells as Markers of Disease Severity in Early and Established RA

As summarized in Table 1, a number of studies have explored the presence of MCs in the synovia of patients with RA. The first observations came from two parallel studies by Crisp et al. and Godfrey et al. The first group analyzed the synovial membrane obtained by either synovectomy or joint replacement from 116 patients with RA. They observed significantly higher MC numbers in RA vs. healthy controls and a correlation of high MC numbers with clinically active synovitis (i.e., swollen joints) but not with ESR [32]. In parallel, Godfrey et al. analyzed a smaller cohort of 14 patients with RA undergoing synoviectomy or joint replacement, again demonstrating higher MC numbers in RA vs. healthy controls [33]. After a few years, another group assessed MCs in the synovia obtained by knee arthroscopy from 20 patients with RA, demonstrating a correlation with synovial inflammation and, interestingly, a reduction of MC numbers after intra-articular steroids in a subset of patients [34]. Accordingly, the study by Tetlow et al. showed increased numbers of MCs in the synovial membrane of RA patients (*n* = 26). For the first time, this group also looked at MC heterogeneity, demonstrating a higher prevalence of MC_T (see Section 3.1 for a detailed discussion on MC heterogeneity) [35]. Overall, in these studies, no attempt to correlate MCs with clinical disease activity was made, and an important limitation is the inclusion of patients with long-standing disease, therefore biased by treatment, which, as directly demonstrated by Tetlow et al., can significantly affect MC numbers in synovia. Gotis-Graham et al. observed an association of synovial MCs with disease activity in 16 patients with established RA. More specifically, the authors observed that MC_TC, a subset of MCs expressing both tryptase and chymase, is expanded in RA and correlates with parameters of disease activity and progression [36]. In a subsequent study, the same authors analyzed the synovial tissue obtained by arthroscopy from 6 patients with early RA (mean disease duration 8 months), demonstrating that early in the course of disease the expansion of MCs is predominantly of the MC_T subset, expressing only tryptase, thus suggesting that different MC subsets might have distinct functions in early vs. late RA [37]. Overall, the results of these studies support the relevance of MCs in the pathogenesis of RA, but the limitations due to disease duration, treatment, and the small number of patients made the interpretation of these results very difficult. It is also important that the above studies did not take into account the clinical and histological heterogeneity of RA, which has emerged in recent years as an important aspect of RA etiopathogenesis [2]. It would be intriguing to speculate that MCs have distinct functions in different patient subsets, and, possibly, at different disease stages. Unfortunately, while there have been many attempts to understand the contribution MCs to arthritis using animal models or in vitro experiments [23], to our knowledge for almost 20 years no other study has attempted to analyze MCs in the synovia of RA patients. The development of ultrasound-guided biopsies has helped to overcome such limitations, allowing the study of synovial inflammation early in the course of the disease [2]. Ramirez et al., by analyzing synovial biopsies from patients with RA in remission, demonstrated that the numbers of synovial MCs and B cells were significantly higher in patients who did not maintain remission after one year [38]. More recently, our group has analyzed the presence of MCs in the synovia of a large cohort (*n* = 99) of patients with early, untreated RA, demonstrating a correlation of MC numbers with local synovitis but also systemic inflammation and disease activity. Importantly, the analysis of synovial MCs allowed for the stratification of patients into groups (low, medium, and high synovial MCs) with different levels of disease activity at baseline [31]. Overall, these data suggest that the analysis of synovial MCs could be used as a biomarker to predict disease severity, progression, and, hopefully, treatment response, although confirmation in larger cohorts and the analysis of longitudinal data pre-post treatment will be needed to further clarify the contribution of MCs to RA and their ability to predict treatment progression and response.

## 4. Concluding Remarks and Future Work

MCs have emerged in recent years as relevant cells in the pathogenesis of RA. Here, we offer a comprehensive review of data on MCs in early RA showing (i) an increase in the numbers of MCs in the synovial membrane of RA patients, (ii) the contribution of MCs to the local inflammatory process via the production of several pro-inflammatory mediators, (iii) the ability of MCs to act as tunable cells by inducing anti-inflammatory responses, and (iv) the correlation of synovial MCs with local and systemic inflammation and disease inflammation. MC key interactions with other immune cells in synovia and their effector functions are summarized in Figure 2. Overall, the evidence presented in this review support a role for MCs in the pathogenesis of RA. Moreover, the data from patients, summarized in Table 1, suggests that the analysis of synovial MCs could help to stratify RA patients. However, an outstanding question remains, whether MCs could be used as markers of disease progression and treatment response. Interestingly, some of the most recent targeted therapies in RA, small molecules inhibiting Janus kinases signaling, have the potential to inhibit the activation of MCs [4], although it is difficult to dissect the specific effects of such treatments on MCs vs. other immune cells. As for their potential relevance as markers of disease progression and treatment response, future analysis of synovial MCs in well-powered biopsy-driven clinical trials exploring the use of synovial histology for patient stratification to different therapies (http://www.r4ra-nihr.whri.qmul.ac.uk and http://www.matura-mrc.whri.qmul.ac.ukwill) hopefully answer such questions soon.

## Figures and Tables

**Table 1:** Summary of the studies analyzing synovial MCs in RA patients.

Year	Title	Disease Duration	Procedure (Joint Site)	Patients (n)	Main Results
Crisp et al. 1984 [32]	Articular mastocytosis in rheumatoid arthritis	Long-standing	Synoviectomy (wrist and knee) and joint replacement (knee)	116	Higher MC numbers in RA vs. healthy samples. Correlation with synovitis but not with ESR. Caveat treatment.
Godfrey et al. 1985 [33]	Mast Cells in Rheumatoid Arthritis and Other Rheumatic Diseases	Long-standing	Synoviectomy or joint replacement	14	Higher MC numbers in RA. Association with clinically active disease.
Malone et al. 1987 [34]	Mast cell numbers in rheumatoid synovial tissues.	Long-standing	Knee arthroscopy	20	Correlation with synovial inflammation. No attempt to look for clinical correlations. Reduction of MC numbers after IA steroids.
Tetlow & Woolley 1995 [35]	Distribution, activation and tryptase/chymase phenotype of mast cells in the rheumatoid	Long-standing	Joint replacement (knee)	26	MC present in all samples, but more abundant in 60% of patients. Higher prevalence of MC_T. No attempt to look for clinical correlations.
Gotis-Graham & McNeil 1997 [36]	Mast cell responses in rheumatoid synovium. Association of the MCTC subset with matrix turnover and clinical progression	Long-standing	Joint replacement or arthroscopy (knee)	16	Increase of MCs in RA vs. OA or healthy samples; MC_TC correlate with disease activity.
Gotis-Graham et al. 1998 [37]	Synovial mast cell responses during clinical improvement in early rheumatoid arthritis	Early (mean disease duration 8 months), sDMARD-naïve	Arthroscopy (knee)	6	Relative increase of MC_T was observed, in correlation with synovial inflammatory score.
Ramírez et al. 2016 [38]	Immunopathologic characterization of ultrasound-defined synovitis in rheumatoid arthritis patients in clinical remission.	Long standing; 20 patients in remission; 22 with active disease	US-guided synovial biopsies	42	MCs and B cells in patients in clinical remission associated with disease reactivation.
Rivellese et al. 2018 [31]	Mast cells in early rheumatoid arthritis associate with disease severity and support B cell autoantibody production	Early (<12 months), steroid and sDMARD-naive	US-guided synovial biopsies (60% small joints, i.e., MCPs or wrists)	99	Synovial MCs are associated with (i) synovial inflammation, (ii) lympho-myeloid infiltrate, (iii) systemic inflammation, (iv) autoantibodies, and (v) disease activity.

^1^ sDMARDs: synthetic disease modifying antirheumatic drugs; MC_T: MC expressing tryptase; MC_TC: MC expressing tryptase and chymase; MCP: metacarpophalangeal, n: number of patients.

## Abbreviations

ACPAs	anti-citrullinated protein antibodies
CRP C	reactive protein
DAPI	4′,6-diamidino-2-phenylindole
ESR	erytrocyte sedimentation rate
FcεRI	Fc epsilon receptor I
MC	mast cell
MC_TC	mast cell expressing tryptase and chymase
MC_T	mast cell expressing tryptase
RANK	receptor activator of nuclear factor kappa-β
RANKL	receptor activator of nuclear factor kappa-β ligand
SCF	stem cell factor
sDMARDs	synthetic disease modifying anti-rheumatic drugs
TLR	toll-like receptors
TNFα	tumor necrosis factor alpha
VEGF	vascular endothelial growth factor
